# Measuring the Economic Impacts of a Circular Economy: an Evaluation of Indicators

**DOI:** 10.1007/s43615-022-00190-w

**Published:** 2022-08-14

**Authors:** A. Kulakovskaya, C. Knoeri, F. Radke, N. U. Blum

**Affiliations:** Chair for Sustainability and Technology, ETH Zurich, D-MTEC Zurich, Switzerland

**Keywords:** Circular economy, Indicator, Value chain, Economic impact, Life cycle thinking, Evaluation

## Abstract

A circular economy (CE) is often seen as a promising way to address pressing environmental challenges, such as climate change, biodiversity loss, and resource depletion. However, the CE concept remains contested, and the implementation of circular strategies (CS) does not automatically improve all dimensions of sustainability. However, assessing the economic impacts of CS implementation is crucial to making the transition from linear to circular value chains. Despite the broad literature on CE indicators, a critical evaluation of economic CE indicators (eCEis) that conduct assessments on a value-chain level is still missing. This study addresses this gap by critically evaluating how capable eCEis are of measuring the economic impacts of implementing CS at the value-chain level. We first identify existing meso eCEis through a literature review, deriving a sample of 13 meso eCEis. We then qualitatively evaluate the eCEis based on criteria synthesised from requirements for CE indicators proposed in the literature. We find that existing meso eCEis only partly fulfil these criteria and consequently have limited capabilities for measuring the economic impacts of the implementation of CS at a value-chain level. The indicators largely satisfy the specific criteria *diagnostic* and *useful*, moderately satisfy the criterion *practical*, and barely satisfy the criteria *systemic* and *transparent*. We therefore recommend that future studies on eCEis place a stronger focus on adopting a systemic perspective, discuss their limitations and uncertainties in more detail, and consider combining meso eCEis with the indicators of other dimensions (environmental, social) and levels (micro, macro).

**Supplementary Information:**

The online version contains supplementary material available at 10.1007/s43615-022-00190-w.

## Introduction

Humankind’s growing impact on the global environment is unequivocal [1–3]. Global greenhouse gas emissions, biodiversity loss, and water stress from resource extraction continue to increase [3–5], while the global consumption of materials and waste generation could almost double by 2050 compared to 2016 [3]. A circular economy (CE), where resource consumption and waste are minimised [6] without compromising or decoupled from long-term economic competitiveness [4], is often seen as a promising way to address these environmental challenges and achieve sustainable development [7–10]. However, the CE concept remains highly contested [6, 10] and has multiple limitations related, for example, to system boundaries, path dependency, thermodynamics, governance, and management [11]. Moreover, implementing a CE through so-called circular strategies (CSs), such as *reduce*, *reuse*, or *recycle* [6], does not automatically have a positive impact on sustainability [12–14]. On the contrary, there exist trade-offs, or burden shifts, between the circulation of materials and the different dimensions of sustainability, namely environmental, economic, and social [9, 14]. It is therefore important to evaluate the impacts of CS implementation in depth, and across multiple dimensions of sustainability, and this study contributes to this goal by focusing on the economic dimension of a CE.

The importance of economic CE research has been broadly recognised by numerous scholars, industries, and institutions alike [10, 15–18], as it remains key for a transition towards more CE. Any CSs, such as *reduce*, *reuse*, or *recycle*, that does not lead to economic benefits for companies under the current regulatory conditions (and is thereby economically not sustainable) is unlikely to be implemented, even if it reduces environmental impacts and should therefore be environmentally desirable [19]. Hence, it is important to identify economically viable CSs to facilitate the transition towards more CE, where the transition starting point is the current economic system and where potentially political intervention is needed to make environmentally beneficial CSs economically attractive.

To systematically monitor the transition, the EU and researchers stress the importance of developing CE indicators [4, 20, 21] that quantify “the changes connected to an intervention” [22]—such as, for example, the economic impacts of implementing CSs. However, studies on economic CE indicators (eCEis) are scarce, and a comprehensive review of such studies is missing. Over the last couple of years, many review studies on CE indicators have been published, such as Pascale et al. [23], Rossi et al. [24], Kristensen and Mosgaard [25], Corona et al. [9], Moraga et al. [26], Parchomenko et al. [17], Sassanelli et al. [27], and Saidani et al. [10]. However, the majority of existing review studies focus on environmental or multi-dimensional CE indicators, whereas the economic dimension remains generally understudied [9, 17, 28]. Notably, no study to date has investigated eCEis specifically despite their high relevance for CE research.

Furthermore, several scholars [10, 28–30] have demonstrated that the majority of CE studies develop CE indicators on the micro (products, companies, consumers) [7, 31] or macro (city, region, nation, and beyond) [7, 10, 32] levels, whereas the meso (eco-industrial parks, value/supply chains) [10, 27, 32, 33] level indicators are generally rarer^1^ despite the importance of addressing CE at the value-chain level has been broadly highlighted in research. Scholars argue that considering the value chain as a whole, instead of looking solely at its individual parts, is essential for closing or slowing (e.g. by extending product lifetimes) material loops [34] and “may result in the creation of a considerably greater resource efficiency potential” [21]. Moreover, the assessment of the impacts (and specifically economic impacts) of implementing CSs is particularly important at the value-chain level because CSs can have diverse effects on different value-chain players [30, 35, 36]. For example, on-site recycling in a manufacturing company can cut material purchasing costs and might also reduce sales for the material supplier, causing their economic losses. Analogously, the introduction of shared mobility or housing can be economically attractive for customers and sharing platforms but negatively affect carmakers or housebuilders. Hence, we must identify the financial winners and losers across the value chain to consider distributional impacts when evaluating economic incentives to foster the transition towards circular value chains.

In summary, despite the broad literature on CE indicators and the multitude of CE indicators across different levels, the evaluation of eCEis at the meso level is still missing. Therefore, we aim to fill this research gap by critically evaluating the applicability of meso eCEis to circular value chains, that is, the value chains in which one or more CSs are implemented. The goal of the evaluation is to identify how well the selected meso eCEis fulfil their purpose, i.e., how capable they are of measuring the economic impacts of implementing CSs at the value-chain level.

The paper is structured as follows. The “Theoretical Background” section introduces the theoretical background and outlines key terms and concepts. The “Methodology” section describes the methodology for (i) collecting CE indicators and identifying therefrom meso eCEis, (ii) developing the evaluation criteria for meso eCEis, and (iii) applying the criteria to the selected meso eCEis. The “Results” section presents the major findings, while the “Discussion” section discusses them, outlining our recommendations for the development of meso eCEis and providing conclusions.

## Theoretical Background

This section outlines the definition of a CE and thereby specifies our connotation of *economic impact* and *meso level*. We further elaborate on the concept of CS introduced by Potting et al. [6] and conclude by selecting and formulating definitions related to *CE indicators*.

The definitions of a CE are numerous [7, 9, 10, 21, 26], yet their boundaries remain blurred [26] and they often neglect crucial elements of a CE, such as the waste hierarchy or linkages to sustainable development [7]. This study adopts the definition by Kirchherr et al. [7], who analysed 114 definitions of CE and suggested that a CE is “circular economy describes an economic system that is based on business models which replace the ‘end-of-life’ concept with reducing, alternatively reusing, recycling and recovering materials in production/distribution and consumption processes, thus operating at the micro level (products, companies, consumers), meso level (eco-industrial parks) and macro level (city, region, nation and beyond), with the aim to accomplish sustainable development, which implies creating environmental quality, economic prosperity and social equity, to the benefit of current and future generations”.

The reasons for selecting this definition are three-fold. First, it incorporates the waste hierarchy, which is important for differentiating between various CSs such as *reduce*, *reuse*, or *recycle*. Second, it has a direct link to the notion of sustainable development that encompasses three dimensions—economic, environmental, and social—and thus makes it possible to better position the economic focus of our study. Lastly, it distinguishes between three levels of analysis (micro, meso, and macro), in which we specifically address the meso level.

The definitions of *micro* and *macro* suggested by Kirchherr et al. [7] are closely comparable with the definitions from other CE studies, such as Ghisellini et al. [31], Saidani et al. [10], and Sauvé et al. [32]. However, researchers often extend the definition of the *meso* level beyond eco-industrial parks. Several scholars [10, 27, 32, 33] refer to the *meso* level as the value (or supply) chain, in addition to eco-industrial parks, and we adopt this extended definition, thus referring to *meso* as both eco-industrial parks and value (supply) chains. Based on Pascale et al. [23], an eco-industrial park in this study is understood as “a group of firms settled within an area that tries to enhance economic, environmental, and social efficiency under reciprocal collaboration, with the aim to generate a greater common advantage than the summation of the single advantages that firms would obtain without cooperation”. A value chain, in turn, is denoted based on Porter [37] as a collection of interdependent activities encompassing multiple stakeholders (companies, organisations) that, for example, produce, deliver, reuse, refurbish, and recycle products or services, and can be settled within a single geographical area or spread beyond it. Thus, both eco-industrial parks and value chains are characterised by multiple interacting stakeholders; however, they can differ with regard to the degree of collaboration (higher for eco-industrial parks) and geographical span (broader for value chains).

In addition to the “meso level”, another key term of this study is *circular strategy*, which can be interpreted as a measure or consciously intended course of action [38] that helps to reduce the consumption of natural resources and materials, as well as minimise production waste [6]. Potting et al. [6] suggest a framework of 10 CSs that can be ordered according to their circularity levels and thus form the waste hierarchy [7]. Circularity levels are highest for those CSs that foster smarter product use and manufacture (*refuse, rethink, reduce*), whereas the circularity levels of the CSs that extend the lifespan of a product and its parts (*reuse*, *repair*, *refurbish*, *remanufacture*, *repurpose*) are lower. CSs that relate to the useful application of materials (*recycle*, *recover*) are the least preferable option from a CE perspective because, for example, incinerated materials are no longer available for use in other products [6]. Thus, the notion of a CS builds on the CE definition by providing more detailed and structured guidelines on how a CE can be implemented—for example, by *refusing*, *rethinking*, or *reducing*.

If the definition of CE explains *what* a CE is, and CSs provide more detail on *how* it can be implemented, then CE indicators tell us *whether* it is working—that is, they measure circularity and the impacts of implementing CSs. Currently, there is no consensus on the definition of the term “indicator” in the CE literature [10, 28, 39]. It is often used synonymously or in conjunction with the terms “index”, “measure”, “metric” [10], “assessment tool”, or “assessment framework” [9]. Given the lack of standardised terminology related to CE assessment, it is important to clarify how key terms are defined and utilised in each CE analysis. We adopt the definition of an indicator proposed by the OECD, as it best serves the purposes of this study. According to the OECD, an *indicator* is a “quantitative or qualitative factor or variable that provides a simple, and reliable, means to measure achievement, to reflect the changes [in this study: economic impacts] connected to an intervention [in this study: implementation of CSs], or to help assess the performance of a development actor” [22]. Henceforth, we use the term “CE indicator” to refer to a quantitative or qualitative method, namely, indicator, index, measure, or metric, to assess the impacts (economic, environmental, and social) of the implementation of CSs. We use the term “CE assessment framework” to describe a methodological framework, for example, life cycle assessment (LCA), life cycle costing (LCC), or material flow analysis (MFA), that can be used to calculate a CE indicator [9]. Subsequently, we define an “eCEi” as a CE indicator that measures the economic impacts of CS implementation. In what follows, we elaborate the meaning of “economic impacts”, the terms in which they can be measured, and what eCEis focus on, as well as the interconnectedness of eCEi results with the environmental and social aspects of a CE.

The economic impacts of implementing CSs can be associated with cost increase or reduction (e.g. lower material costs as a result of recycling), the gain or loss of economic value (e.g. additional income from selling production waste as an input material), and the comprehensive economic feasibility of CS-related investments (e.g. traditional investment analysis of cash flow generated from investments in recycling or energy-efficiency facilities) (adapted from [40]). As with many other indicators, eCEis can be measured in absolute or relative terms. Absolute indicators can be expressed, for example, as a number (count), cost, or time [25]—for example, the total annual cost of reusing a glass bottle, or additional annual income from selling production waste. Relative indicators measure the “change in a nominal value relative to its value in a reference period” [23] and are usually expressed as a percentage or ratio—for instance, the cost difference between a linear system and one where one or more CSs are implemented.

Furthermore, eCEis can have a single focus (be unidimensional); that is, they only address an economic dimension of sustainability, or they can have a split focus [25] (be multidimensional); that is, they also address other sustainability dimensions, such as environmental and/or social. This is often the case for CE indices that aggregate individual indicators into a single index on the basis of an underlying model of the multi-dimensional concept that is being measured [41]. Lastly, it is important to note that the results of eCEis can have different implications for different sustainability dimensions. For instance, the increase in labour costs from implementing remanufacturing activities can be considered a negative economic impact of implementing CSs at the company level. However, from a social perspective, it can be seen as having a positive impact because new jobs are created. Thus, when evaluating or developing eCEis, it is crucial to distinguish their level (micro, meso, macro), their concrete impact (e.g. cost reduction), their terms and units (e.g. cost, time, percentage), and their focus (single or split)—as well as to discuss the potential implications and trade-offs of eCEi results for the non-economic dimensions of sustainability.

## Methodology

To evaluate existing meso eCEis, we first identified existing meso eCEis through a literature review. Second, we derived a set of evaluation criteria from the literature. Third, we assessed the identified meso eCEis.

### Identification of Meso Economic CE Indicators

**Fig. 1:**
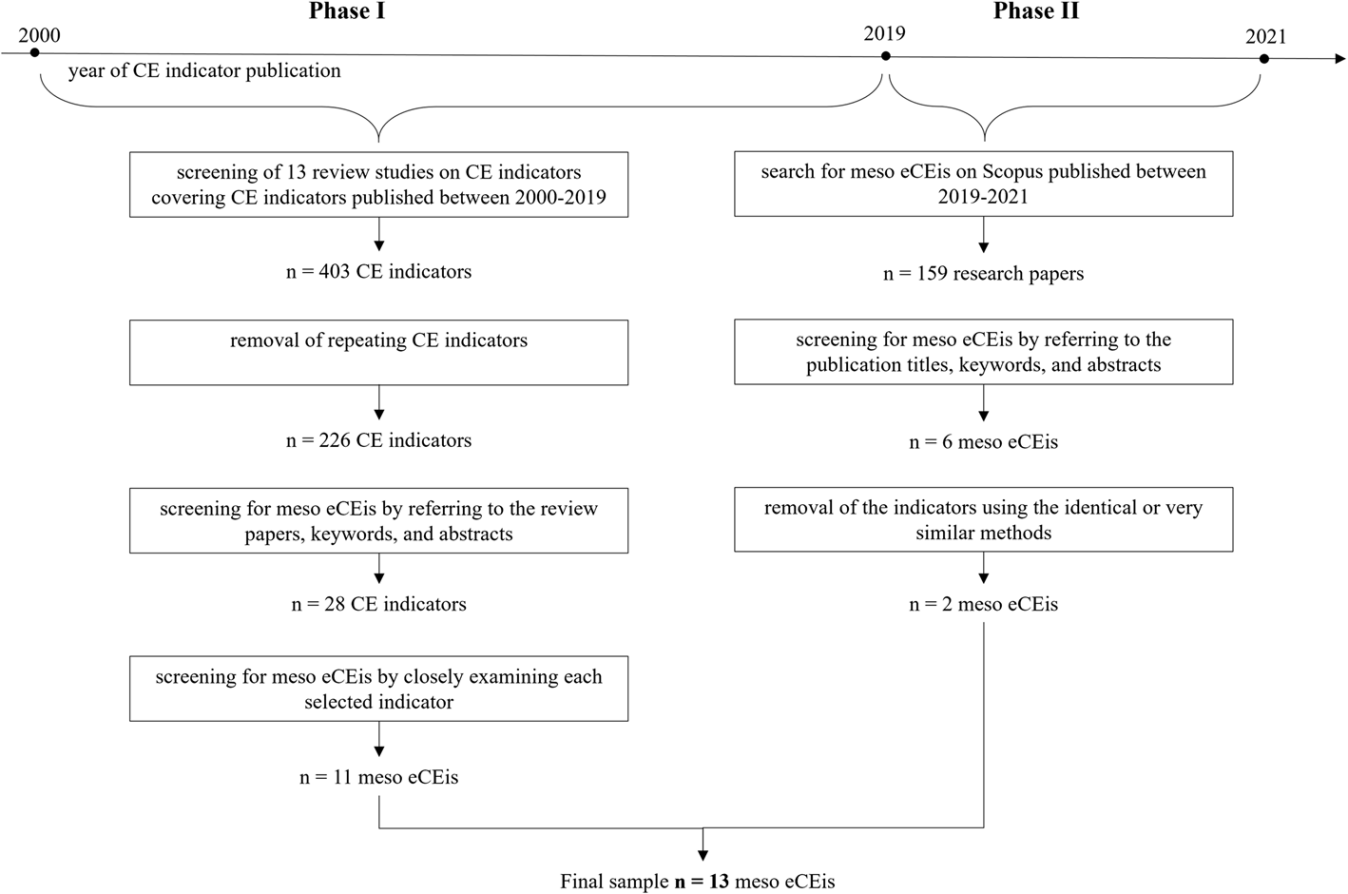
Scheme summarising the process of identifying existing meso eCEis

To derive a list of meso eCEis, we adhered a two-phase literature review approach. In phase I, we screened existing review studies for meso eCEis, i.e., conducted a meta-analysis, which represents a useful method for summarising and integrating results from different studies on similar research topics [42]. In phase II, we complemented the identified meso eCEis from the meta-analysis with additional literature search to account for recent years that were not covered in the review studies. The procedure for selecting meso eCEis is summarised in Fig. 1 and described in detail below.

#### Phase I

The meta-analysis of existing review studies for meso eCEis was conducted in four steps. First, we selected recent review studies that systematically list, compare, categorise, and evaluate CE indicators. Specifically, we considered 13 review studies published between 2017 and 2021 that covered the indicators published in academic journals as well as institutional and industry reports between 2000 and 2019 [9, 10, 17, 20, 23–27, 30, 33, 43, 44] (Table 1). Thus, we identified 403 CE indicators across different levels (micro, meso, macro) that assess either one specific sustainability dimension (economic, environmental, or social) or several dimensions simultaneously.

**Table 1:** Overview of CE indicators (*n* = 403) based on the selected review studies 2017–2021

	Review study author(s)	Publication year	Number of reviewed indicators
1	Linder et al. [33]	2017	5
2	Elia et al. [30]	2017	16
3	Pauliuk [20]	2018	28
4	Walker et al. [43]	2018	8
5	Moraga et al. [26]	2019	20
6	Saidani et al. [10]	2019	55
7	Parchomenko et al. [17]	2019	63
8	Corona et al. [9]	2019	16
9	Helander et al. [44]	2019	10
10	Sassanelli et al. [27]	2019	61
11	Kristensen and Mosgaard [25]	2020	30
12	Rossi et al. [24]	2020	30
13	Pascale et al. [23]	2021	61
	Total number of CE indicators		403

Second, we excluded repetitions, which yielded a total of 226 CE indicators, and then screened them for the type (economic) and level (meso) by referring to the classifications from the selected review papers. In cases where the classifications by type and/or level were not provided, we searched for specific keywords and considered the abstracts of the original CE indicator publications.^2^ This resulted in 28 eCEis. Lastly, we closely examined each selected CE indicator to ensure that it provided information sufficient for our assessment and was aligned with our definition of meso eCEis. As a result, we excluded five indicators proposed by the industry, as the information necessary for the assessment was not sufficient or not publicly available (e.g. Assessing Circular Trade-offs (ACT) by [45], Circularity Indicator Project (ICT) by [46]). We also excluded 12 indicators proposed by scholars, since they are not in line with our selected definition of eCEis—such indicators represent broad analytical frameworks (e.g. [47–49]) or do not directly measure economic impacts (e.g. energy-based indicators by [50] or [51]). Thus, 17 meso eCEis in total were excluded from the sample, and 11 were selected for the final assessment.

#### Phase II

As the considered review studies covered publication years up to 2019, we conducted an additional academic literature^3^ search for meso eCEis published between 2019 and 2021.^4^ The search resulted in 159 research papers, which were further filtered by reading the titles, keywords, and abstracts of each paper. We excluded studies that conducted policy analysis, proposed a broad evaluation framework, focused on the environmental or social dimensions of sustainability, analysed innovation and design strategies, or conducted an assessment on a product, company, country, or global level. The screening resulted in six meso eCEis. Some of these eCEis used identical or very similar assessment methods, such as hybrid life cycle assessment [52–54] or cost–benefit analysis [55–57], but applied them to different case studies. Having removed such repetitions, we selected two additional meso eCEis for the final assessment, thus yielding 13 meso eCEis in the final sample.

### Development of the Evaluation Criteria for Meso Economic CE Indicators 

Multiple review studies, as well as institutional and industry reports on CE, have suggested several overarching requirements, or evaluation criteria for CE indicators, which can be used to assess how far indicators fulfil their purpose [9, 10, 20, 33, 58, 59]. Such criteria include, for instance, *construct validity*, *transparency*, and *generality* [33], or *systemic by nature*, *integrated*, and *operational* [10]. To assess how far the selected meso eCEis fulfil the purpose of measuring the economic impacts of CSs’ implementation, we synthesised the criteria for CE indicators proposed in the literature and adapted them to the economic dimension. We proceeded in three steps: First, we derived the *general criteria*—validity, reliability, and utility—based on the criteria suggested by Corona et al. [9], Linder et al. [33], Parchomenko et al. [17], Bannigan and Watson [60], and Park and Kremer [61]. Second, we used these general criteria to derive a set of more specific criteria: *systemic*, *diagnostic*, *consistent*, *transparent*, *robust*, *practical*, and *useful*. Lastly, we developed definitions for these specific criteria by synthesising the relevant definitions from the literature and adapting them to the economic dimension. Table 2 summarises the developed *general* and *specific* criteria for meso eCEis with the definitions, followed by an explanation of their development and adaptation processes (a more detailed explanation is presented in Table 6 in the Appendix).

**Table 2:** General criteria (GC) and specific criteria (SC) with definitions for the evaluation of meso eCEis

GC	SC	Definitions
Validity	Systemic	Adopts a LCT approach, that is, considers the economic impacts of CS implementation across the entire life cycle of products or services, including pre-production, production, use, and end-of-use [21, 26]
		Distinguishes different CSs, the implementation of which may result in economic impacts, instead of focusing on one particular CS such as recycling [21, 33]
	Diagnostic	Identifies causation (cause-effect relationships), that is, measures the economic impacts of CS implementation [62, 63]
		Shows improvement (alternatively, progress, development, or benchmark) in economic impacts over time [10, 58, 64]
Reliability	Consistent & transparent	Gives the same result on separate occasions, for example, if used repeatedly by different stakeholders (e.g. researchers, policymakers, or practitioners), and is reproducible [60]
		Transparent; that is, it comes with a clear description of the process of selection, development, and application, can be verified by third parties, and extensively describes limitations (e.g. data-related, methodological) and the risks of unintended consequences [33, 60, 65, 66]
	Robust	Gives the same result independently from minor changes or errors in the model [33, 62, 65, 67]
		Based on internationally standardised and established CE assessment frameworks (e.g. LCA, MFA, LCC, economic MFA) [68]
Utility	Practical	Feasible in terms of data input, that is, the data from relevant stakeholders can be collected and analysed at a reasonable cost [21, 61, 62]
		Flexible, that is, can be applied in various industries and for various products or services [9]
	Useful	Addresses the needs of stakeholders, that is, can serve the economic objectives of industrial, institutional, or political decision-makers [62, 63]
		Simple (intuitive) and can be easily communicated, for example, in a single aggregated value [62, 69]

Validity, according to Corona et al. [9] and Linder et al. [33], relates to the degree that an indicator measures what it is supposed to measure, implying in the context of this study the extent to which the indicator measures economic impacts of CS implementation at a system level, where the system encompasses an entire value chain. Thus, we argue that a *valid eCEi* must be both *systemic* and *diagnostic*. *Systemic* (or “systemic by design”), according to Saidani et al. [21] and Moraga et al. [26], implies that the indicator (i) adopts a Life Cycle Thinking (LCT) approach, which, in the context of this study, considers the processes that are part of the life cycle of a product or service and that are related to the economic impacts. Furthermore, a s*ystemic* indicator, according to Saidani et al. [21], (ii) distinguishes between different CSs instead of focusing on one particular strategy, such as recycling. The importance of a systemic perspective has been emphasised by other researchers—for example, Pauliuk [20] outlines that “there is a general understanding that both CE and sustainable business practice require a systems perspective on the role of businesses in the wider system of stakeholders and the environment”. Moraga et al. (2019) [26] argue that “several reviews on CE show the necessity of a systemic view of the life cycle of resources”, and Parchomenko et al. (2019) [17] state that “the assessment of value maintenance on a system level, or on an integrated product-system level, is currently poorly addressed, and should thus be improved as to contribute to the validity of the CE concept, representing a key aspect of the CE”—thus reinforcing the relevance of considering the *systemic* criterion as part of the validity of the CE.

*Diagnostic*, in turn, describes a CE indicator that (i) can identify cause–effect relationships [62, 63], that is, measures the economic impact from CS implementation, and (ii) reflects improvements over time [58, 64], or, alternatively, provides information by tracking progress, providing a benchmark, or identifying areas of improvement [10] that are related to economic impacts.

Reliability, according to Corona et al. [9], relates to the *consistency* and *robustness* of the indicator; for example, it gives the same results across different practitioners or occasions, and is *transparent*, which is in line with the definition provided by Linder et al. [33] who suggested that a *reliable* indicator should give similar values under consistent conditions. Thus, we suggest that a reliable indicator should be *consistent*, *transparent*, and *robust*.

*Consistency*, according to Bannigan and Watson [60], refers to the stability of an indicator—that is, how far it will give the same results on separate occasions (for example, when it is used repeatedly by different stakeholders, such as researchers, policymakers, or practitioners)—and is closely linked to the notion of reproducibility [60, 62].

Transparency implies that the indicator comes with a clear description of the process of selection, development, and application [65], can be verified by third parties [33], and fully describes limitations (e.g. methodological, data-related) and risks of unintended consequences [66].

*Robustness* is characterised by numerous definitions in the literature that describes it, for example, as the ability to withstand stresses, pressures, or changes in procedure or circumstance [70]; make the subjective or normative elements of evaluation explicit; be reproducible, non-perverse [62], and less error-prone [67]; use statistically validated/quality data [62, 68]; and adhere to internationally established and standardised methodologies [68]. The relevance of this last point has been further emphasised by Borrion et al. [71], Corona et al. [9], Moraga et al. [26], Linder et al. [33], and Walker et al. [43], who advocate constructing CE indicators based on well-established methodologies or CE assessment frameworks such as LCA and/or MFA. Thus, to avoid overlaps with the criterion *consistent* and integrate recommendations from CE research, we propose that a *robust* metric (i) shows the same result independently from minor changes or errors in the model and (ii) is based on internationally standardised and established CE assessment frameworks, such as LCA and/or MFA.

The last general criterion—utility—relates, according to Corona et al. [9], to the *practicality* of an indicator. Park and Kremer [61] extend this definition, suggesting that a high-utility metric must also be *useful*. Building upon these findings, we suggest that a high-utility metric is *useful* and *practical*.

The difference between *practicality* and *usefulness* is subtle, as can be seen from the following connotations, yet we attempt to clarify it by suggesting concrete definitions. Saidani et al. [21] associate *practicality* with the two “mandatory and required features” of a CE indicator: being integrated into industrial practices and being operational, that is, being able to gather “adequate” data and “support data construction”. In a similar fashion, Atlee and Kirchain [62] link practicality to operationalisation, whereas Corona et al. [9] relate it to flexibility and ease of implementation, thus aligning relatively closely with Park and Kremer [61], who define practicality as the “perceived cost and time to learn and to implement an indicator”. Drawing upon these connotations, we suggest that a *practical* indicator must be (i) operational, that is, it is feasible in terms of data input (data can be collected and analysed at a reasonable cost), and (ii) flexible, suggesting that the indicator can be applied in various industries and can assess various products or services. Such an interpretation of flexibility supports the recommendations of Cayzer et al. [69], who encourage the development of CE indicators for “different industry sectors and product types”, and Linder et al. [33] who invite us to “explore the possibility of cheaper and more lightweight approximations of circularity in various industries”.

While *practicality* can be linked to the implementation of an indicator in terms of data input, *usefulness* is instead related, according to Atlee and Kirchain [62] and Oswald [63], to its theoretical simplicity, as well as to the goals, needs, and objectives of the stakeholders who implement it. Thus, Atlee and Kirchain [62] propose that a *useful* metric addresses a clear goal and is simple/specific (user-friendly^5^), diagnostic, and comparable. They further argue that “the usefulness of a measure depends on the needs of the stakeholders using the metric”, and that an ideal metric is “useful at all levels, could be aggregated, and [is] valuable for cross comparisons as well as real-time decision making”. Oswald [63] supports the stakeholder-oriented interpretation of *usefulness*, claiming that “metrics are a tool for corporations to improve performance and measure progress towards set targets. Before defining metrics, the objectives of the activity need to be well defined and clear so that the metric can be tailored to the objectives”. Based on these proposals, we suggest that a *useful* metric (i) addresses the needs of stakeholders, that is, can serve the economic objectives of industrial, institutional, or political decision makers, and (ii) is simple (intuitive) and can be easily communicated, for example, in a single aggregated value. To summarise, the developed specific evaluation criteria for an eCEi inferred from validity, reliability, and utility include *systemic*, *diagnostic*, *consistent*, *transparent*, *robust*, *useful*, and *practical* (Table 2); their detailed derivation steps are presented in Table 6 in the Appendix.

### Application of the Specific Criteria to the Selected Meso Economic CE Indicators

We applied the specific criteria to the selected meso eCEis to evaluate their capability to measure the economic impacts of CS implementation at a value-chain level. We qualitatively evaluated whether the indicators satisfied each part of the criteria definition (“yes”, “partly”, “no”, and “N/A”) by referring to the original publications that proposed the selected meso eCEis. For example, we considered the criteria *systemic* as fully satisfied (assigned “yes”) if both parts of its definition—“adopts LCT approach” and “distinguishes different CSs”—are satisfied. However, if only one part of the definition was satisfied, we considered the criteria *systemic* as only partly satisfied (assigned “partly”). The rules for assigning “yes”, “partly”, “no”, and “N/A” to criteria definitions are specified in Table 3, whereas the application of the criteria to the selected meso eCEis is detailed in supporting information.

**Table 3:** Rules for assigning “yes”, “partly”, “no”, and “N/A” to criteria definitions

	Criteria definition	Rule
Systemic	Adopts LCT approach	**Yes** = 4 life cycle phases are covered (pre-production, production, use, and EOU) **Partly** = 3 life cycle phases are covered **No** = 0–2 life cycle phases are covered
	Distinguishes different CSs	**Yes** = more than 3 CSs are distinguished **Partly** = 2–3 CSs are distinguished **No** = 0–1 CS is distinguished
Diagnostic	Identifies causation	**Yes** = the indicator measures an economic impact and it is clear how the impact changes depending on the implementation of a particular CS **Partly** = the indicator measures an economic impact, however, it is unclear how the impact changes depending on the implementation of a particular CSs **No** = the indicator does not measure an economic impact
	Shows improvement over time	**Yes** = the indicator shows improvements over time, or, alternatively, tracks progress, provides a benchmark, or identifies areas of improvement that are related to economic impacts **Partly** = the indicator identifies areas of improvement, but it does not show improvements over time, track progress, or provide a benchmark **No** = the indicator does not identify areas of improvement and does not show improvements over time, track progress, or provide a benchmark
Consistent & transparent	Gives the same result on separate occasions	**Yes** = the indicator gives the same result on separate occasions, e.g. if used repeatedly by different stakeholders (e.g. researchers, policymakers, or practitioners) **Partly** = the indicator gives slightly different results (within one standard deviation of the mean in a normal distribution) on separate occasions, e.g. if used repeatedly by different stakeholders (e.g. researchers, policymakers, or practitioners) **No** = the indicator gives different results (outside one standard deviation of the mean in a normal distribution) results on separate occasions, e.g. if used repeatedly by different stakeholders (e.g. researchers, policymakers, or practitioners) **N/A** = sensitivity analysis and datasets are not provided, and therefore it is not feasible to assess the consistency of the results
	Transparent	**Yes** = the indicator comes with a clear description of an indicator development and application, and extensively discusses its limitations and/or uncertainties (e.g. data-related, methodological) **Partly** = the indicator comes with a clear description of an indicator development and application, however, the discussion of its limitations and/or uncertainties is rather incomprehensive or absent **No** = the indicator does not come with a clear description of an indicator development and application, and the discussion of its limitations and/or uncertainties is rather incomprehensive or absent
Robust	Result is independent from minor changes or errors	**Yes** = the indicator shows the same result independently from minor changes or errors in the model **Partly** = the indicator shows slightly different results (within one standard deviation of the mean in a normal distribution) depending on minor changes or errors in the model **No** = the indicator shows different results (outside one standard deviation of the mean in a normal distribution) depending on minor changes or errors in the model **N/A** = sensitivity analysis and datasets are not provided, and therefore it is not feasible to assess the robustness of the results
	Based on an established CE assessment framework	**Yes** = the indicator uses LCA- or MFA-based assessment frameworks (e.g. LCA, LCC, MFA, MFCA) as main methodology **Partly** = the indicator uses LCA- or MFA-based assessment frameworks as additional or secondary methodology **No** = the indicator does not use LCA- or MFA-based assessment frameworks **N/A** = the information on methodology is not provided
Practical	Feasible in terms of data input	**Yes** = the indicator can be calculated with the data from publicly available databases and/or literature sources that are free of charge (e.g. governmental databases, open access academic journals or reports) **Partly** = the indicator can be calculated with the data from paid databases and literature sources (e.g. Ecoinvent database, non-open access academic journals or reports), and/or the data from study participants (e.g. through qualitative interviews) **No** = the indicator can be calculated only with the data collected from study participants
	Flexible	**Yes** = the indicator can be applied in various industries and for various products and services **Partly** = the indicator can be applied in various industries, but not for various products and services, or vice versa **No** = the indicator cannot be applied in various industries and for various products and services
Useful	Addresses the needs of stakeholders	**Yes** = the indicator can address economic needs or objectives of stakeholders and clearly specifies its target audience (e.g. industrial, institutional, or political decision-makers) **Partly** = the indicator can address economic needs or objectives of stakeholders, however, it does not clearly specify its target audience **No** = the indicator cannot address the economic needs or objectives of any stakeholders
	Simple/intuitive	**Yes** = the logic of the indicator is simple/intuitive, and the result is communicated in a single value **Partly** = the logic of the indicator is simple/intuitive; however, the result is not communicated in a single value; *or*, *conversely*, the result is communicated in a single value, however, the logic of the indicator is rather complex and/or non-intuitive **No** = the logic of the indicator is rather complex and/or non-intuitive, and the result is not communicated in a single value **N/A** = formula(s) and method(s) used are not provided, therefore, no conclusions about the logic and result can be drawn

As a final note, it is important to mention that the current study, which suggests multiple criteria definitions, does not seek to evaluate eCEis against all the suggested definitions. Specifically, two criteria definitions—“gives the same result on separate occasions, reproducible” (consistent) and “gives the same result independently from errors” (robust)—are included in Table 2, which summarises the criteria; however, they are excluded from Table 5, where the selected meso eCEis are evaluated. This is because evaluation based on these two criteria definitions would require access to many different data sets and an extensive statistical analysis of each indicator, which is neither provided in the selected meso eCEis studies nor included in the scope of this work. Although the definitions of these two criteria cannot be used for the current *evaluation*, they are nevertheless essential for the *development* of any indicator, and hence are important from a theoretical perspective.

## Results

This section presents the results of the evaluation of the selected meso eCEis based on the developed criteria. First, we list the selected meso eCEis with a short description in Table 4, and then we outline the evaluation results, summarising them in Table 5.

**Table 4:** Selected meso eCEis

#	Abbr	Full name	Author	Source (A/O)	Dimension (M/S)	Meso sub-level (EIP/VC)	Short description
1	HLCA	Hybrid life cycle assessment	Chen et al. [52]	A	M	VC	The method evaluates and optimises the sustainability of supply chain through a hybrid life cycle assessment model. The model considers the economic (gross value added), social (employment hours), and environmental (GHG emissions) impacts using the example of potato pulp valorisation in the biocomposite supply chains (adapted from [52])
2	EPOS	Enhanced energy and resource Efficiency and Performance in process industry Operations via onsite and cross-sectorial Symbiosis	Cervo et al. [56]	A	M	EIP	The EPOS methodology is developed in the framework of the H2020 European project EPOS with the objective of enabling cross-sectoral industrial symbiosis (IS). EPOS methodology aims at facilitating the preliminary assessment, the engagement of stakeholders, the identification of IS opportunities, and the definition of feasibility. It defines *inter alia* synergy scenarios and assesses them economically through cost–benefit analysis based on seven economic indicators: GDP, GDP growth, public benefits, private benefits, reinvestment, economic weight (% of territorial turnover, % of territorial added), and economic performance (adapted from [56])
3	WCI	Wastewater Circonomics Index	Kayal et al. [72]	A	M	EIP	The Circonomics Index measures the circularity of wastewater industry. The component indicators of the index are linked directly to the three Rs: reduce, reuse, and recycle. Specifically, the index is a product of wastewater production efficiency indicator (for *reduce*), composite wastewater reuse indicator (for *reuse*), and wastewater recycling indicator (for *recycle*). These indicators consider various monetary terms, such as average selling prices, total annual costs, or shadow prices (adapted from [72])
4	EEIX	Eco-efficiency IndeX	Laso et al. [73]	A	M	VC	The indicator links the environmental performance of a product to its economic value by combining LCA and LCC (economic value added) assessments. The LCA-LCC results are coupled with linear programming tools to derive a composite eco-efficiency index. The economic part of the study measures (i) cost reductions by calculating the costs of raw materials, pre-processing, processing, and manufacturing, primary and secondary packaging, and waste treatment; and (ii) value added by calculating the difference between total incomes and costs of bought-in materials and services (adapted from [73])
5	BWM	Best–Worst Method	Zhao et al. [74]	A	M	EIP	The method evaluates the benefit of eco-industrial parks in terms of circular economy and sustainability. It proposes an index system that includes economic, social, and environmental benefit criteria with nine quantitative sub-criteria and four qualitative sub-criteria. The key economic benefit sub-criteria include the annual average growth rate of industrial added value (quantitative), the proportion of research and development input value in GDP (quantitative), and the correlation degree of enterprises (qualitative). As a next step, a new comparison-based method, the best–worst method, was employed to determine the weights of all sub-criteria and the performance values of all selected eco-industrial parks with respect to the qualitative sub-criteria (adapted from [74])
6	CETUS	Circular Economy Toolbox US	US Chamber of Commerce Foundation [75]	O	M	VC	Toolbox of 11 CE indicators, where two indicators—Estimated Cost Savings per Rental (ECSR) and Return on investment (ROI)—are related to economic impacts and therefore assessed jointly. ECSR measures what an individual saves when leasing or renting versus buying a new product. ROI (usually in %) expresses the profit made for a set investment: (net profit/cost of investment) × 100. Often used with investments in capital equipment, manufacturing facilities, or infrastructure to support circular economy models (adapted from [75])
7	VRE	Value-based Resource Efficiency	Di Maio et al. [67]	A	U	VC	The indicator assesses resource efficiency and circular economy in terms of the market value of ‘stressed’ resources. Specifically, it focuses on the value of non-sustainable/stressed inputs to the economy relative to output. The input is what traditional industry sectors use, such as energy, raw materials, labour, and semi-finished components. The output is the value added of the economy /industry /sector. As an example, in an ideal case, a circular economy uses sustainable resources, such as renewable inputs from the bio-sphere, upcycled components, and recycled wastes that have low prices/values per kilogram. It uses as little non-sustainable/stressed inputs as possible, and creates jobs and a high value added (adapted from [67])
8	EEP	Eco-Efficiency Performance	Pagotto and Halog [76]	A	M	VC	The study used two complementary input–output (IO)-oriented approaches to analyse the economic and environmental efficiency performance of the Australian food system subsectors. First, the research involved the analysis of the required inputs for the entire food supply chain in Australia using material flow analysis (MFA). The environmental impacts caused by the food supply chain were evaluated and analysed. Next, the research reported the calculated eco-efficiency—that is, the economic and environmental efficiency performance of various subsectors in the Australian food system—using data envelopment analysis (DEA). For the economic evaluation, the value added to the economy was used (adapted from [76])
9	RPI	Resource Productivity Indicator	Wen and Meng [77]	A	M	EIP	The method combines the substance flow analysis (SFA) approach with the resource productivity (RP) indicator to evaluate the contribution of industrial symbiosis (IS) to the development of circular economy (adapted from [77])
10	EEIR	Eco-Efficiency IndicatoR	Park and Behera [78]	A	M	EIP	The eco-efficiency indicator is proposed as an integral parameter for simultaneously quantifying the economic and environmental performance of industrial symbiosis (IS) networks. It includes one economic indicator (net economic benefit) and three simplified environmental indicators (raw material consumption, energy consumption, and CO2 emissions) (adapted from [78])
11	FCIM	Five Category Index Method	Li and Su [79]	A	M	EIP	The five-category indicator, where one category—economic development—is related to economic impacts. It measures the rate of output per unit of land area, per capita GDP, rate of return on common stockholders’ equity, and annual growth rate of industrial added value. Other categories are environment-related and measure resources exploiting, pollution reduction (reducing effluents), biological efficiency, and developmental potential. The indicator is used to assess the circularity level of Chinese chemical industries (adapted from [79])
12	MIND	Method for analysis of Industrial energy systems	Karlsson and Wolf [80]	A	M	EIP	A model comprising a pulp mill, a sawmill, a district heating network, and a biofuel upgrading plant is used to demonstrate how the MIND method, an optimisation method based on mixed integer linear programming that can be used to evaluate industrial symbiosis in the forest industry. The optimisation is performed by taking the cost into consideration. The total system costs of an integrated system with a chemical pulp mill, a sawmill, and a biofuel upgrading plant are compared to a system with similar stand-alone plants (adapted from [80])
13	QAEEA	Quantitative Assessment of Economic and Environmental Aspects	Jacobsen [81]	A	M	EIP	The method evaluates environmental and economic aspects of the selected water-related exchanges within an industrial symbiosis (IS). The economic aspects of the IS projects are estimated and discussed as a combination of direct cost reductions, real investments in relation to alternative avoided investment scenarios, and estimated payback times for the different exchange projects at the time of project initiation (adapted from [81])

Legend: A=academia, O=other organisation, M=multidimensional, U=unidimensional, EIP=eco-industrial park, VC=value chain.

**Table 5:** The results of the analysis of selected meso eCEis based on the general and specific evaluation criteria. Legend: 1 = “yes” (criteria definition is fully satisfied);—= “partly” (partly satisfied); 0 = “no” (not satisfied); N/A = information for the criteria definition was not attainable, and hence it cannot be evaluated. The rules for assigning “yes”, “partly”, “no”, and “N/A” to the evaluation criteria are specified in Table 3

Indicator abbreviation	Source	Validity				Reliability		Utility			
		Systemic		Diagnostic		Transparent	Robust	Practical		Useful	
		Adopts LCT approach	Distinguishes different CSs	Identifies causation	Shows improvement over time	Describes development, application, limitations, uncertainties	Based on an established CE assessment framework	Feasible in terms of data input	Flexible, that is, can be applied in various industries and for various products or services	Addresses the needs of stakeholders	Its logic is simple/intuitive; easily communicates a result
HLCA	[52]	0	0	1	1	-	1	-	1	1	1
EPOS	[56]	0	1	-	1	1	0	-	1	1	-
WCI	[72]	0	-	1	1	-	0	1	0	1	1
EEIX	[73]	0	1	1	1	1	1	-	1	1	1
BWM	[74]	N/A	0	-	1	-	0	-	1	1	1
CETUS	[75]	0	0	1	1	0	0	-	1	-	1
VRE	[67]	-	-	-	1	-	0	1	1	1	1
EEP	[76]	1	-	-	1	-	-	-	1	1	1
RPI	[77]	0	1	-	1	-	1	-	1	1	1
EEIR	[78]	0	1	1	1	1	0	-	1	1	1
FCIM	[79]	0	0	-	1	-	0	-	1	-	1
MIND	[80]	0	0	-	1	1	0	-	1	1	-
QAEEA	[81]	0	-	-	1	-	0	-	1	1	1

### Meso Economic CE Indicators

We identified 13 meso eCEis developed between 2006 and 2020 (Table 3). The majority—12 out of 13 eCEis—was developed by academia, except for the Circular Economy Toolbox US (CETUS) proposed by the non-profit association U.S. Chamber of Commerce Foundation. Similarly, 12 out of 13 selected eCEis—except value-based resource efficiency (VRE)—are multidimensional, indicating that they assess not only economic but also other dimension(s) of sustainability. Five eCEis—hybrid life cycle assessment (HLCA), Eco-Efficiency Index (EEIX), CETUS, VRE, and eco-efficiency performance (EEP)—assess the economic impacts at the value-chain level, whereas the other eight address them at the level of eco-industrial parks.

### Evaluation of the Selected Meso Economic CE Indicators

The results of the evaluation obtained through the application of the developed specific and general criteria for meso eCEis are summarised in Table 5. They generally show that the selected eCEis largely satisfy the specific criteria *diagnostic* and *useful*, moderately satisfy the criterion *practical*, and barely satisfy the criteria *systemic* and *transparent.* In the following section, we outline the results of each evaluation criterion in greater detail.

The results show that the selected eCEis generally do not, or only partly, fulfil the validity criterion. Namely, according to the developed criteria, the selected eCEis do not (fully) satisfy the criterion *systemic* and hence are not capable of successfully measuring the economic impacts of CS implementation at a system level, where the system encompasses an entire value chain—even though the importance of adopting a systemic perspective has been broadly emphasised in research (e.g. by Moraga et al. [26], Parchomenko et al. [17], Saidani et al. [10], and Pauliuk [20]). Specifically, the selected eCEis fall short of adopting an LCT approach by considering the economic impacts of CS implementation across the entire life cycle of products or services. They also distinguish three or less different CSs, focusing primarily on *reduce*, *reuse*, and *recycle*, and neglecting several other CSs, such as *rethink*, *repair*, or *refurbish*—except the indicators EEIX, RPI, and EPOS that consider, for example, *reduce*, *reuse*, *recycle*, *repurpose*, or *recover*. However, all selected eCEis demonstrate better performance in terms of the criterion *diagnostic*: they successfully manage to identify cause-effect relationships—that is, to measure the economic impacts of CS implementation and show improvements over time—or, alternatively, track progress, provide a benchmark, or identify areas of improvement that are related to economic impacts.

The analysis across reliability criteria demonstrates that, according to the *robust* criterion, only three indicators (HLCA, EEIX, and RPI) are based on an established CE assessment framework, such as LCA or LCC, whereas the other meso eCEis propose rather idiosyncratic methodologies for indicator calculation, thus making it challenging to harmonise various CE indicators. The analysis of the eCEis for the criteria *transparent* shows that the majority of the indicators are well documented; that is, they include a clear description of an indicator’s development and application. However, they do not extensively discuss their limitations and/or uncertainties, and hence can be considered partly transparent in contrast to EPOS, EEIX, EEIR, and MIND, which provide an extensive discussion of their shortcomings and thus fully satisfy the transparency criterion.

Lastly, the eCEis generally demonstrate medium to high performance in terms of the utility criteria *practical* and *useful*. Many of the indicators can be applied in various industries and for various products or services and thus can be considered flexible––except WCI, which specifically targets the wastewater industry. They also successfully address stakeholders’ needs and objectives, and communicate results in a single aggregated value with a simple and intuitive logic behind it. However, almost all eCEis require a relatively costly data-collection process, which impedes their feasibility in terms of data input. Most of the eCEis, apart from VRE and WCI, cannot be calculated with data from publicly available databases and/or literature sources that are free of charge (e.g. governmental databases, open-access academic journals or reports). They usually require data from paid databases and literature sources (e.g. Ecoinvent database, restricted-access academic journals, or reports) and/or data from study participants (e.g. value-chain stakeholders).

## Discussion

In this section, we discuss, first, the implications for researchers that relate to the improvement of the existing eCEis and the contribution of our findings for CE indicators in general. Second, we highlight the relevance of our findings for policymakers and industry by discussing the application of meso eCEis and highlighting the importance of combining them with other CE indicators.

### Implications for Research

Our evaluation of meso eCEis demonstrated that the selected existing indicators are generally *diagnostic* and *useful*, moderately *practical*, but only partly *systemic*, *transparent*, and *robust.* Previous review studies on CE indicators [9, 17, 21, 33, 44] similarly found that the existing CE indicators do not fulfil these criteria, or lack specific features they propose. In contrast to our study, the cited reviews often had a wider scope, as they (i) consider multiple dimensions of sustainability (i.e. environmental, economic, social); (ii) define the criteria, desired qualities, or features of CE indicators differently; and (iii) do not focus exclusively on the meso level, considering various levels of analysis (micro, meso, macro), or do not differentiate CE indicators based on an analysis level. Thus, despite the structural and content differences between the present study and previous review studies, our results generally support the finding that existing CE indicators are not fully capable of fulfilling their purposes. To better identify their gaps and suggest how future research can enhance the capability of meso eCEis, we focus on the only partly- or non-satisfied specific criteria *practical*, *systemic*, *transparent*, *and robust* in the following.

The criterion *practical* is only moderately satisfied due to low data feasibility. Whereas the majority of the indicators proved to be applicable in various industries and for various products, only a few indicators show high feasibility in terms of data input. The calculation process of the selected indicators often requires data from paid databases and literature sources and/or the data from study participants. This underlines the need to make data more transparent and accessible to make eCEis, and potentially all other CE indicators, more practical.

The majority of the indicators do not, or only partly, satisfy the criterion *systemic*. This can be explained by the fact that many meso eCEis assess the economic impacts at the level of eco-industrial parks, and since such parks by definition have a narrower geographical scope than value chains, they are less likely to contain firms with activities covering an entire product life cycle. However, several meso eCEis that evaluate the impacts at a value-chain level also do not always adopt an LCT approach, as they usually consider the pre-production and production phases and neglect the use and end-of-use phases, despite the latter might be having significant economic impacts. Furthermore, several meso eCEis are not fully systemic, as they distinguish only three or less different CSs, which demonstrates a relatively narrow perspective on CE that focuses mostly on *reduce*, *reuse*, and *recycle*. Thus, we recommend that future indicators expand their scope by first considering the economic impacts of CS implementation across the entire life cycle of products or services, instead of focusing on a few life cycle phases. This might be more feasible to achieve if meso eCEis assess the impacts at a broader value-chain level than at the level of eco-industrial parks. Second, we recommend that eCEis capture and ideally be capable of assessing more than three CSs (e.g. *refuse*, *rethink*, *repair*, *refurbish*, *remanufacture*, *repurpose*, *recover*), as this is vital for ensuring a holistic perspective on CE.

Similarly to *systemic*, the criterion *transparent* is satisfied only to a certain extent, as the limitations and/or uncertainties (e.g. data-related, methodological) of the selected eCEis are rarely discussed in detail. This impedes a nuanced understanding of the indicators and makes it difficult to estimate whether and to what extent they are reliable. Thus, we encourage future studies to discuss their shortcomings in greater scope and depth, as a comprehensive discussion of indicators’ shortcomings is crucial to enhancing the overall transparency and thus the reliability of an indicator.

Furthermore, the criterion *robust* is only partly satisfied, as the majority of the selected meso eCEis do not use established CE assessment frameworks such as LCA, LCC, MFA, or MFCA. Rather, their evaluations are based on other methods, including data envelopment analysis (DEA), input–output analysis (I-O) [76], optimisation method based on mixed integer linear programming (MIND) [80], model with weighted sub-indicators [74, 79], cost–benefit analysis (CBA) [56], or standalone methodologies [67]. Although such methods can prove effective in separate studies, they hardly make it easier to combine and compare CE assessments across a multitude of studies. In other words, despite certain limitations of LCA- and MFA-based methodologies, we recommend that future meso eCEis use them as a basis for assessment, as this may foster their harmonisation and comparability, which is currently lacking yet highly needed in CE research [59]. Furthermore, the more complex the value chain, the more difficult it might be to provide robust and transparently reported data. Hence, the established data reporting standards can be particularly relevant for robust and transparent value-chain CE assessments, which can be further enhanced through, for example, digitalisation or regulatory instruments (e.g. the German Supply Chain “Das Lieferkettengesetz”).

Lastly, we emphasise that the two aspects—“gives the same result on separate occasions, reproducible” (*consistent*) and “gives the same result independently from errors” (part of *robust*)—were not included in the evaluation of the selected eCEis. The evaluation based on these aspects is not feasible within the scope of this study; however, we argue that they must be considered during the development of any CE indicator to enhance its overall reliability. This can be done, for example, by providing applied data samples, performing a sensitivity analysis, and sharing the results, or by using consistent databases from established and trusted sources. Micro indicators might perform better in terms of consistency and robustness, as they tend to have lower uncertainty levels in general compared to meso and macro indicators [67]. Hence, we recommend that future research on CE indicators performs a holistic sensitivity analysis and incorporates publicly available data sources with a robust track record in the development and application of meso eCEis.^6^

Analogously to the two aspects of consistent and robust, also other specific criteria can be generalised to environmental and social sustainability indicators and different indicator levels (micro, meso, macro). We encourage future research developing CE indicators to adhere a systemic and diagnostic perspective, consistency, transparency, robustness, practicality, and usefulness independently of an indicator type and level. This can be done, for example, by adapting the criteria definitions to a specific sustainability dimension, i.e., by specifying the type of impact in the criteria *systemic* and *diagnostic* and the type of objective in the criteria *useful*.

### Implications for Policymakers and Industry

The discussion of the shortcomings of existing meso eCEis helps to better understand how their individual capabilities can be improved. However, the improvement at the level of individual indicators is not sufficient to achieve a holistic perspective on CE and thus foster the transition from linear to circular value chains. The key is to apply meso eCEis in a way that fosters the transition, and this can be done by combining meso eCEis with indicators of other dimensions (environmental, social) and levels (micro, macro), striking the balance between various stakeholders’ interests. In what follows, we explain in more detail how meso eCEis can be applied by policymakers and industry to benefit CE.

First, we suggest combining meso eCEis with indicators that measure other dimensions of sustainability. While meso eCEis provide insights into how economically a circular value chain operates, which still provides value for managers and policymakers, it is not sufficient to evaluate different CSs holistically. To achieve a holistic perspective on CSs, which is key for a successful CE transition, it is important that policymakers assess environmental and, if possible, social sustainability in addition to the economic dimension [14]. This will enable, first, a more nuanced picture of potential externalities that are not reflected in a purely economic assessment relying on prices, but can be captured if a social dimension is considered [82–84]. Second, it can help to identify trade-offs (or burden shifts [9]) between different dimensions of sustainability, i.e., conflicts between environmental, economic, and social objectives [85]. This, in turn, is essential for informing policymakers about potential risks and opportunities associated with an implementation of CSs. The evaluated meso eCEis are predominantly multidimensional; that is, they already integrate multiple dimensions of sustainability and thus successfully adopt a holistic approach to CE. Unidimensional eCEis, which appear to be less prevalent in CE research, can be integrated with environmental and social CE indicators, for example, through the use of the abovementioned established CE methodologies that facilitate the harmonisation and comparability of indicators.

Furthermore, we highlight that meso indicators—alongside macro indicators—are particularly relevant for policymaking. Macro-level indicators extend their scope from environmental and economic dimensions to encompass a social perspective—measuring, for example, employment [10]. Hence, they can give countries an indication of the potential economic gains of transforming their overall economy into a CE and can help to decide on the allocation of, for example, research and development funding for more circular solutions, or to incentivise the reuse of locally scarce resources (e.g. phosphorous recycling in Switzerland [86]). In a similar fashion, indicators on the level of a value chain (meso eCEis that look beyond eco-industrial parks) are useful for identifying more economically beneficial CSs for a specific value chain and are therefore useful for a conglomerate of several companies along a value chain (e.g. CEFLEX, a collaborative initiative representing the entire value chain of flexible packaging [87]), a single country (e.g. with a certain country-internal value chain, such as the wood industry in Switzerland), or a coalition of countries (e.g. the EU) to make entire value chains more circular and sustainable. Hence, meso-level indicators that focus on value chains provide knowledge about value-chain dynamics and can help policymakers to make decisions regarding incentives and resource allocation, and therefore should be considered in policymaking together with macro-level metrics. Micro indicators, in turn, are targeted to the needs of individual companies. They assess the economic and environmental dimensions of sustainability, often focusing on resource flows and end-of-life strategies (particularly recycling) [24, 25] and therefore can be useful for single businesses or organisations to make informed decisions about new products or services.

## Conclusion

This study evaluated meso economic circular economy indicators (meso eCEis) to identify how well they fulfil their purpose—that is, how capable they are of measuring the economic impacts of implementing CSs at a value-chain level. The indicators were evaluated based on the six evaluation criteria (*systemic*, *diagnostic*, *consistent & transparent*, *robust*, *practical*, and *useful*) derived from three more general criteria *validity*, *reliability*, and *utility*. Our evaluation of meso eCEis demonstrated that the selected indicators generally fulfil the specific criteria *diagnostic* and *useful*—they demonstrate good performance in terms of tracking progress, identifying causation, addressing stakeholder needs, and being simple (intuitive). They are also moderately *practical*, as they are capable of measuring different products and services across various industries but are not highly feasible in terms of data input. However, the selected indicator sample falls short of being *systemic*, *transparent*, and *robust*—the indicators hardly adopt a systemic perspective (i.e., consider the entire life cycle of products or services and distinguish different CSs—aspects that are both essential for a value-chain perspective), are not sufficiently transparent, and often do not adopt an established CE assessment framework, such as LCA- or MFA-based methodologies. This provides insight into how meso eCEis can enhance their capability to measure the economic impacts of CS implementation.

However, the improved capability of individual indicators is not enough to effectively foster the transition to circular value chains. It is crucial to combine meso eCEis with indicators that measure other sustainability aspects, particularly environmental and social impacts, to achieve a holistic picture of CE and to identify CSs that contribute to more sustainability overall. It is also paramount to consider them alongside the indicators of other levels, particularly macro indicators. That is, meso eCEis can facilitate decision-making, especially for policymakers, as they complement macro-level indicators. Macro eCEis give countries an indication of the potential economic gains of transforming their overall economy into a CE. By contrast, meso eCEis have the potential to support policymaking for a specific value chain, both within a country (especially if the value chain is located within the country’s borders) or across countries, for example, in the EU, where value chains extend across national borders. This kind of policymaking currently lacks proper eCEis, thus highlighting the need to fill this gap by developing more meso eCEis that satisfy the specified evaluation criteria.

There are several limitations to this study. First, the study abstained from (i) broad assessment frameworks aimed at analysing CE at a value-chain level across multiple dimensions (e.g. [47–49].) and (ii) CE indicators that do not directly measure economic impacts (e.g. energy-based indicators by [50] or [51]). Hence, future studies can extend the evaluation scope by including such frameworks and CE indicators in their analysis, clearly outlining the definition of meso eCEis. Second, a list of the general and specific evaluation criteria for eCEis was developed based on the literature. Thus, the findings could be more profound if additional sources are considered—for example, surveys, or qualitative semi-structured interviews with various stakeholders in one or more industries to reveal their perspectives on the criteria for eCEis. Lastly, the classification of indicator types (economic, environmental, or social) and levels (micro, meso, macro) was not always clear-cut; hence, there might be overlaps across indicator types and levels. It is therefore important for future studies that analyse and/or propose eCEis to clearly define the type and level of their respective indicators to facilitate their evaluation and better position them in CE research.

To conclude, this study has contributed to the ongoing research on CE indicators by exploring the strengths and weaknesses of existing meso eCEis. Our findings shed light on how one can enhance the capability of future indicators and apply them to effectively foster the transition from linear to circular value chains.

### Electronic supplementary material

Below is the link to the electronic supplementary material.

Supplementary file1 (XLSX 33 KB)

## Data Availability

Not applicable.

## Appendix

**Table 6:** Derivation of the evaluation criteria for meso eCEis based on the requirements for CE indicators from the literature

General criteria with definition	Inferred specific criteria	Definitions of the specific criteria in the literature	Definition of the specific criteria for this study
Validity (The degree to which a metric measures what it is intended to measure, implying in the context of this study the extent to which the indicator measures economic impact from CS implementation at a system level, where the system encompasses an entire value chain)	Systemic	“’[*S]ystemic* by design’ cornerstone highlights that the measurement tool should encompass a wide spectrum of the circular economy paradigm—including its complexity and principles. Such as *lifecycle thinking*, consideration of *systemic levels* and *interplay* between implementation *levels* (macro, meso, micro, and nano). […] Additionally, a multi-dimensional scoring system representing different perspectives of circular economy should be preferred, and ideally, *distinction between circularity loops […]* should be established” (Saidani et al. [21], p.13) “CE acts on several steps of the production and consumption chain so that indicators may use a *Life Cycle Thinking (LCT)* approach. LCT is the capacity to look at products or services over the cycles of design, production, consumption, use, and disposal including *interactions with sustainability”* (Moraga et al. [26], p. 454) “[S]everal reviews on CE show the necessity of a *systemic* view of the *life cycle* of resources” (Moraga et al. [26], p. 454)	Adopts a LCT approach, that is, considers the economic impacts of CS implementation across the entire life cycle of products or services, including pre-production, production, use, and end-of-use Distinguishes different CSs, the implementation of which may result in economic impacts, instead of focusing on one particular CS such as recycling
	Diagnostic	“[I]dentification of *cause-effect relationships* that result in different metric values” (Oswald [63], p.19) “To be diagnostic, a metric must facilitate the identification of patterns in metric results, development of hypotheses, and the determination of *causation* that underlies differences in metric values” (Atlee and Kirchain [62], p.4507) “[A]ssess efficiency and continuous *improvement* efforts” (Bouton et al. [58], p.15) “Show *developments* over a relevant time interval go with an explanation of causes behind the trends” (Gabrielsen and Bosch [88], p.5) “[I]nformation purposes, helping to understand the situation (tracking *progress*, providing a *benchmark*, or identifying areas of *improvement*)” (Saidani et al. [10])	Identifies causation (cause-effect relationships), that is, measures the economic impacts of CS implementation Shows improvement (alternatively, progress, development, or benchmark) in economic impacts over time

Reliability (the consistency and robustness of the indicator, e.g. it gives the same results by different practitioners or occasions and is transparent)	Consistent & Transparent	“Reliability (or *consistency*) refers to the *stability* of a measurement scale, i.e. how far it will give the *same results on separate occasions”* (Bannigan and Watson [60], p. 3238) “ ‘[R]eliability is a statistical measure of how reproducible the instrument’s data are’ (Utwin 1995, p. 6) and can be equated with stability, consistency and dependability” (Bannigan and Watson [60], p. 3239) “Hence, we refer to the possibility of third-party verification in terms of the *transparency* of a metric” (Linder et al. [33], p.547) “The process of selection, development and application of indicators and the qualitative, quantitative or descriptive methods of assessing individual indicators shall be capable of being *transparently* reported” (ISO 21929–1 [65], p. 29) “It should be clear and *transparent* about any *limitations* (e.g. data, methodological) and *risks* of unintended consequences” (EU [66], p.27)	Gives the same result on separate occasions, for example, if used repeatedly by different stakeholders (e.g. researchers, policymakers, or practitioners), and is reproducible Transparent; that is, it comes with a clear description of the process of selection, development, and application, can be verified by third parties, and extensively describes limitations (e.g. data-related, methodological) and the risks of unintended consequences
	Robust	“This is quality of being able to *withstand stresses, pressures, or changes* in procedure or circumstance” (Nathan and B. Sudhakara [70], p.278) “Subjective Elements Explicit, Reproducible, Nonperverse, Quality Data Available” (Atlee and Kirchain [62], p. 4506) “[A] robust product-level circularity metric should focus exclusively on measuring circularity as a single attribute of product quality” (Linder et al. [33], p. 546) “Robustness of the indicator is guaranteed by an *international standardised method,* statistically validated data, and sensitive data” (Wisse [68], p. 3) “[L]ess robust (i.e., it is more *error-prone*)” (Di Maio et al. [67], p. 164)	Gives the same result independently from minor changes or errors in the model Based on internationally standardised and established CE assessment frameworks (e.g. LCA, MFA, LCC, economic MFA)
Utility (how practical and useful is the metric)	Practical	“Integrated & *operational*” (defined through ‘practical’)” (Saidani et al. [21], p. 12) “*[F]lexible* and *easy to implement*” (Corona et al. [9], p.3) “Perceived *cost and time* to *learn* and to implement an indicator” (Park and Kremer [61], p. 807) “*Operational* metrics by which to practically assess progress toward sustainability” (Atlee and Kirchain [62], p. 4506) “[T]o be operational, as one of the main challenges to evaluate properly product circularity lies on the ability to *gather adequate date*, the framework should support *data* construction” (Saidani et al. [21], p. 13)	Feasible in terms of data input, i.e. the data from relevant stakeholders can be collected and analysed at a reasonable cost Flexible, that is, can be applied in various industries and for various products or services
	Useful	“Addresses a *clear goal, simple/specific*, diagnostic, and comparable” (Atlee and Kirchain [62], p. 4506) “The usefulness of a measure depends on the *needs of the stakeholders using the metric*. While the ideal metric would be useful at all levels, could be *aggregated*, and be valuable for cross comparisons as well as real-time *decision making*, this is rarely achievable in practice” (Atlee and Kirchain [62], p. 4508) “[M]etrics are a tool for corporations to improve performance and measure progress towards set targets. Before defining metrics, the *objectives* of the activity need to be *well defined* and *clear* so that the metric can be tailored to the objectives” (Oswald [63], p. 17) “A multi-measure approach is taken, with a single aggregated metric for each lifecycle stage. This approach has several advantages: speed, simplicity; ease of diffusion; comprehensible metaphor. However, there are some limitations and challenges: hiding of complexity; potentially misleading results; superficial engagement with decision-making; and the reliance on context-specific assumptions” (Cayzer et al. [69])	Addresses the needs of stakeholders, that is, can serve the economic objectives of industrial, institutional, or political decision-makers Simple (intuitive) and can be easily communicated, for example, in a single aggregated value

## Footnotes

1 The studies that reviewed CE indicators across the three levels of analysis found that meso CE indicators are the least frequent. For example, Banaite[29] identified 65 micro indicators, 46 meso indicators, and 153 macro indicators [20]; Saidani et al. [10] in their taxonomy considered 20 micro, 16 meso, and 19 macro CE indicators; Vinante et al. [28] determined 368 micro, 45 meso, and 183 macro high-granularity CE metrics; and Sassanelli et al. [27] found that CE indicators mostly focus on inter-company level, whereas intra-company level, including urban areas, industrial parks, and supply chains, is less prevalent.

2 To identify the economic indicators from Saidani et al. [10], we searched for the key words “value-based”, “value creation”, “value captured”, “three dimensions of sustainability considered”, “business models”, “financial”, “economic”, and “cost savings”, and selected the indicators that are described with these key words. To identify economic indicators from Parchomenko et al. [17], we searched for the CE elements “value change”, “retention”, “supply risk”, and “longevity”, and selected the indicators that are plotted close to these elements. To identify meso indicators from the pre-determined pool of economic indicators from Sassanelli et al. [27], we searched for the key words “industrial park”, “supply chain”, and “value chain”, and selected the indicators that are described with these key words. To identify meso indicators from the pre-determined pool of economic indicators from Corona et al. [9] and Walker et al. [43], we considered the abstracts of the original CE indicator publications.

3 Although we acknowledge the contribution of meso eCEis developed outside of academia, within this study, we solely concentrated on academic literature as, supposedly, they are developed independent of economic or political interests. Furthermore, the indicators proposed outside academia, for example, by consulting companies, NGOs, or governmental bodies, often do not publicly disclose the minimum information necessary for current evaluation, or/and focus on micro (businesses) level, such as CIRCelligence by the Boston Consulting Group 2021 or Circulytics by the Ellen MacArthur Foundation 2020.

4 We searched in the Scopus database within article titles, abstracts, and keywords for the terms “circular economy”, “indicator”, “index”, “measure”, “metric”, “assessment”, “economic”, “cost”, “revenue”, “value added”, “financial”, “life cycle costing”, “meso”, “value chain”, “supply chain”, “industrial symbiosis”, “eco-industrial park”, and “economic system”, limiting our search to final articles in English published between 2019 and 2021.

5 Alternatively, “intuitive” [21].

6 The evaluation of the existing meso eCEis with the criteria derived from the literature provided a structured assessment of their performance. However, when developing new meso (and potentially micro and macro) eCEis, there might be additional criteria that should be fulfilled. The existing CE indicator literature suggests such additional criteria relate to 1) product and material features (quality [9, 26]; scarcity [9]; lifespan [33]); 2) methodological and computational issues (assessment of open loops; number of time a product/service is provided [9]; function-preservation strategies [26]; burden-shifting [9]; distinction between circularity and impacts [9, 10]; external costs [33]); 3) particular economic aspects (production costs [33]; employment; rapid price fluctuations [33]; consumer behavior [9]); and 4) other aspects (importance of collaborative research [33] and sector-specific indicators [10]). We suggest that when developing an eCEi, these criteria should be considered in addition to the specific evaluation criteria introduced in this study.
